# Effects of chronic cholinergic stimulation associated with aerobic physical training on cardiac morphofunctional and autonomic parameters in spontaneously hypertensive rats

**DOI:** 10.1038/s41598-021-96505-2

**Published:** 2021-08-25

**Authors:** Camila B. Gardim, Ana Catarine Veiga, Bruno A. Aguilar, Stella V. Philbois, Hugo C. D. Souza

**Affiliations:** Ribeirão Preto Medical School, University of São Paulo, Ribeirão Preto, SP Brazil

**Keywords:** Physiology, Cardiology, Diseases

## Abstract

We investigated hemodynamic, cardiac morphofunctional, and cardiovascular autonomic adaptations in spontaneously hypertensive rats (SHRs) after aerobic physical training associated with chronic cholinergic stimulation. Fifty-four SHRs were divided into two groups: trained and untrained. Each group was further subdivided into three smaller groups: vehicle, treated with pyridostigmine bromide at 5 mg/kg/day, and treated with pyridostigmine bromide at 15 mg/kg/day. The following protocols were assessed: echocardiography, autonomic double pharmacological blockade, heart rate variability (HRV), blood pressure variability (BPV), and baroreflex sensitivity (BRS). Physical training and pyridostigmine bromide reduced BP and HR and increased vagal participation in cardiac autonomic tonic balance. The associated responses were then potentialized. Treatment with pyridostigmine bromide increased HRV oscillation of both low frequency (LF: 0.2–0.75 Hz) and high frequency (HF: 0.75–3 Hz). However, the association with physical training attenuated HF oscillations. Additionally, treatment with pyridostigmine bromide also increased LF oscillations of BPV. Both treatment groups promoted morphofunctional adaptations, and associated increased ejection volume, ejection fraction, cardiac output, and cardiac index. In conclusion, the association of pyridostigmine bromide and physical training promoted greater benefits in hemodynamic parameters and increased vagal influence on cardiac autonomic tonic balance. Nonetheless, treatment with pyridostigmine bromide alone seems to negatively affect BPV and the association of treatment negatively influences HRV.

## Introduction

Systemic arterial hypertension is accompanied by important cardiovascular autonomic impairments, which include a decrease in baroreflex sensitivity (BRS), heart rate variability (HRV), and blood pressure variability (BPV)^1,2^. These autonomic impairments are associated with cardiovascular morphological and functional changes, and when they are not identified and treated promptly, they may contribute to the development of heart failure, reducing quality of life and life expectancy.

Thus, the search for new pharmacological and non-pharmacological therapies with cardioprotective actions is of great relevance to the prevention and/or reversal of the development of hypertension^3–5^. In this light, clinical and experimental studies have demonstrated that pyridostigmine bromide, a drug regularly used to treat myasthenia gravis, promotes cardiac and cardiovascular autonomic benefits by reducing heart rate (HR) at rest^6,7^ and enhancing HRV^8–11^. On the other hand, lifestyle modifications such as regular physical exercise also promote important benefits in cardiovascular autonomic control and contribute to positive cardiac remodelling^4,12^.

In fact, some cholinergic stimulation effects of pyridostigmine bromide on cardiac autonomic control are similar to those observed in aerobic physical training as observed in both human and experimental models^5,10,13,14^. An experimental study in spontaneously hypertensive rats (SHRs) showed that cholinergic stimulation with pyridostigmine bromide for 2 weeks reduced blood pressure (BP) and HR. However, ejection fraction, a parameter evaluated by radioisotopic ventriculography, was also reduced in this study. Additionally, autonomic parameters showed an increase in vagal participation in autonomic tonic balance and a decrease in systolic BPV^9^.

Although the hemodynamic and autonomic improvements observed are important and suggest a beneficial effect, the reduction of ejection fraction (EF) is worrying. This may be related to the pyridostigmine bromide dosage used in the study (25 mg/kg/day), or perhaps to the methodology employed to investigate EF. However, with only one functional parameter evaluated, it was not possible to precisely determine the effects of pyridostigmine bromide on cardiac function. In this case, we hypothesized that treatment with low doses of pyridostigmine bromide at 5 mg/kg/day and 15 mg/kg/day in SHRs would promote positive hemodynamic and cardiovascular autonomic effects as well as changes in morphological and functional cardiac parameters without affecting EF. In turn, the association with aerobic physical training will promote a catalytic effect, possibly increasing gains obtained with pyridostigmine bromide.

## Methods

### Animals and procedures

**Figure 1:**
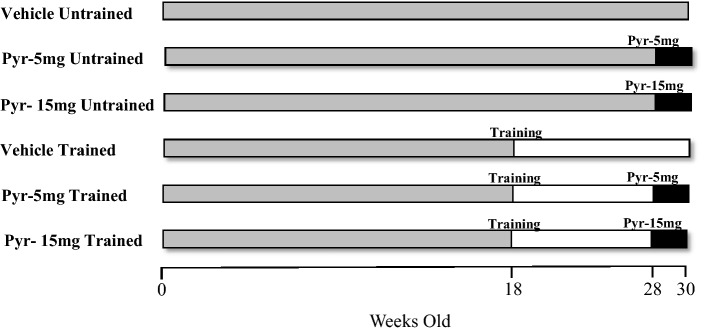
Schematic representation of the experimental timeline of all studied groups.

Fifty-four 18-week-old SHRs were divided into two treatment groups: untrained (n = 27) and trained (n = 27). Each group was further subdivided into three smaller groups (n = 9): vehicle (H_2_O), pyridostigmine bromide (Sigma-Aldrich, Saint Louis, MO, USA) diluted in drinking water at a dose of approximately 5 mg/kg/day, and pyridostigmine bromide diluted in drinking water at a dose of approximately 15 mg/kg/day. Ingestion was measured and corrected every day using graduated drinking fountains. For the two treatment groups, pyridostigmine bromide was given for 2 weeks, spanning the 11th and 12th weeks of aerobic physical training (Fig. 1). The doses were determined based on the results of a previous study^9^. During the experiments, the animals were housed at the Animal Facility of the Ribeirão Preto Medical School, which was maintained at 23 °C and 60–70% humidity. The rats were kept on a 12/12-h light/dark cycle and had free access to food and water. The experimental protocols used in the present study were in accordance with the ethical principles of animal experimentation adopted by the Brazilian College of Animal Experimentation and were evaluated and approved by the Animal Experimentation Ethics Committee (CETEA) of the Ribeirao Preto Medical School, University of Sao Paulo (Protocol 035/2014). This study was carried out in compliance with the ARRIVE guidelines.

### Physical training

The SHRs in the training groups underwent a protocol of aerobic physical training that consisted of swimming sessions in a glass tank (100 cm long × 80 cm wide × 80 cm high), which allowed for the simultaneous training of six animals. The tank was filled with 50 cm of warm water (30 ± 2 °C), which was changed after every group training session. The training program was conducted in two different stages over a total of 12 weeks, covering 18 to 30 weeks of age of the SHRs. The first stage consisted of a 2-week adaptation period, during which the session length was gradually increased from 5 to 30 min per day (in increments of 5 min per day), five times per week. The second stage consisted of 10 weeks of 30-min physical training sessions conducted five times per week^15^. To evaluate physical training intensity, blood was collected from the tail veins of the animals at the fourth, seventh, and 10th weeks of physical training immediately before and after the 30-min exercise sessions, and lactate concentration was measured (Accutrend^®^ Plus, Roche Diagnostics, Mannheim, Germany). The expected lactate level ranged from 5.5 to 6 mmol/L as previously determined. If the SHRs did not achieve the expected lactate concentration, the level of training exertion was increased by fastening a leaded, impermeable Velcro strap to the chest to increase body weight by 2–6%^16^.

### Echocardiography

At 30 weeks of age, all animals underwent an echocardiographic evaluation. We used a Vevo 2100^®^ High-Resolution Imaging System ultrasound (VisualSonics, Toronto, ON, Canada) instrument with a high-resolution transducer (21 MHz). For the procedure, the anterior regions of the thorax were previously trichotomized (Veet^®^, Reckitt Benckiser, São Paulo, SP, Brazil), and all animals were anesthetized with 1.5% isoflurane supplemented with 1% O_2_ and placed on a heated (37 °C) platform. Echocardiography and temperature measurements were also performed.

High-resolution B-mode and M-mode images were acquired. Wall thickness and left ventricle dimensions were obtained from a short-axis view at the level of the papillary muscles. Diastolic measurements were performed at the point of maximum cavity dimension, and systolic measurements were performed at the point of minimal cavity dimension. All measurements were performed according to the standards of the American Society of Echocardiography and by an evaluator who was blinded to which group the rats were assigned at the time of measurement^17^. The following parameters were obtained from the images: interventricular septum thickness (IVST), posterior wall thickness (PWT), end-diastolic diameter of the left ventricle (LVEDD), and end-systolic diameter of the left ventricle (LVESD). The shortening fraction was calculated with the following equation: FS lrb% = [(LVEDD-LVESD ÷ LVEDD) × 100. EF was calculated using the Teichholz method: (LVEDV-LVESV ÷ LVEDV) × 100. The left ventricular mass (LV mass/final body weight) was calculated using the following formula: 1.047 × [(LVEDD + PWT + IVST)^3^ − LVEDD^3^], and the relative wall thickness (RWT) was calculated as follows: [2 × PWT ÷ LVEDD]. Left ventricular volumes were quantified using the following formula: LVEDV (µL) = [LVEDD^3^ × (7 ÷ 2.4 + LVEDD^3^)] and LVESV (µL) = [LVESD^3^ × (7 ÷ 2.4 + LVESD^3^)]^18,19^.

### Surgical procedure

Fourty eight hours after echocardiography, polyethylene catheters (PE-10/PE-50, Intramedic; Becton Dickinson and Company, Sparks, MD, USA) were implanted into the left femoral artery and vein of the SHRs under ketamine (5 mg/kg, intraperitoneal [i.p.]; Sigma-Aldrich, USA) and xylazine (30 mg/kg, intraperitoneal [i.p.]; Sigma-Aldrich, USA) anesthesia in order to record pulsatile BP and administer drugs. The catheters were subcutaneously tunneled and exteriorized in the nape. Twenty-four hours after the surgical procedures for implementation of catheters into the left femoral artery and vein, the pulsatile AP was measured in conscious rats kept in a quiet environment. The AP was recorded using a pressure transducer (MLT0380; AD Instruments, Bella Vista, Australia), and the amplified signal (ML110; AD Instruments, Bella Vista, Australia) was fed to a computer acquisition system (PowerLab 8/30; AD Instruments, Bella Vista, Australia). The systolic arterial pressure (SBP), mean arterial pressure (MBP) and heart rate (HR) were calculated from the pulsatile AP.

### Experimental protocols—autonomic evaluation

#### Heart rate (pulse interval) variability and systolic blood pressure variability

The analysis of pulse interval (PI) and AP variability was performed from the basal recordings using a custom-made computer software (CardioSeries-v2.4, http://sites.google.com/site/cardioseries)^20,21^. The systolic BP and PI of series obtained from 60-min recordings were converted to data points every 100 ms using cubic spline interpolation and were divided into half-overlapping sequential sets of 512 data points (51.2 s). All the segments were visually inspected, and the nonstationary data were discarded^22–24^. A Hanning window was used to attenuate the side effects, and the spectrum of each segment was computed using a direct Fast Fourier Transform (FFT). The spectra were integrated in low (LF: 0.2–0.75 Hz) and high frequency bands (HF: 0.75–3.0 Hz), and the results were expressed in absolute (ms^2^ or mmHg^2^) and normalized units (nu). The normalized values were created by calculating the percentage of LF and HF power of the total spectrum power minus the very low-frequency band (VLF: < 0.2 Hz)^25^. To assess the sympathovagal balance, the LF/HF ratio of the PI variability was calculated^26^.

#### Spontaneous baroreflex sensitivity

Baroreflex sensitivity (BRS) was assessed in the time domain using the sequence technique described by Di Rienzo et al*.*^27^. A custom computer software (CardioSeries v2.4, http://sites.google.com/site/cardioseries) was used to scan the beat-by-beat time series of SBP and PI, searching for sequences of at least four consecutive beats in which increases in SBP were followed by PI lengthening (up sequence) and decreases in SBP were followed by PI shortening (down sequence) with a linear correlation higher than 0.8. The slope of the linear regression lines between SBP and PI was used as a measure of spontaneous BRS.

#### Assessment of cardiac sympathovagal balance

The influence of sympathetic and parasympathetic autonomic tone on HR was assessed by administering propranolol (5 mg/kg, intravenous [i.v.], Sigma-Aldrich, USA) and methylatropine (4 mg/kg, i.v.; Sigma-Aldrich, USA) to the SHRs, respectively. For this purpose, the femoral artery catheter of the SHRs was attached to a pressure transducer (MLT844, AD Instruments, Bella Vista, Australia), which converts AP fluctuations into electrical signals. Signals were then amplified using a bridge amplifier (FE117, AD Instruments, Bella Vista, Australia), and pulsatile AP was continuously sampled (2 kHz) using a computer equipped with an analog–digital interface (ML866, AD Instruments, Bella Vista, Australia). After 60 min of basal HR recording, methylatropine was injected into half of the SHRs in each group, and HR was recorded for the following 15 min to assess the effect of vagal blockade on HR. Propranolol was then injected into the same SHRs, and HR was recorded for another 15 min to determine intrinsic HR (IHR). In the remaining half of the SHRs in each group, the methylatropine–propranolol sequence was reversed to assess the effect of sympathetic blockade on HR, following the same recording procedure (15 min each) for each drug to determine the IHR. The data from methylatropine–propranolol and propranolol**–**methylatropine sequences were pooled to provide the basal HR (i.e., before any drugs) and the IHR (i.e., after drugs).

### Statistical analysis

The results are presented as mean ± standard error of the mean (SEM). The effects of hypertension and pharmacological treatments were assessed using two-way analysis of variance (ANOVA). When appropriate, posthoc comparisons were performed using the Student-Newman–Keuls test. For comparison between two groups, the Student’s t-test for independent measures or the Mann–Whitney Rank Sum test was used as required. Differences were considered significant at P < 0.05. All statistical tests were performed using SigmaPlot 11.0 software (Systat Software Inc., San Jose, CA, USA; https://systatsoftware.com).

## Results

### Baseline parameters

Table 1 shows the baseline parameters, the treatment with pyridostigmine bromide at a dosage of 15 mg/kg/day, associated with aerobic physical training, promoted an increase in body weight. Both aerobic physical training and pyridostigmine bromide reduced BP and HR values. When associated with aerobic physical training, the reduction in SBP and diastolic BP (DBP) was more prominent.

**Table 1:** Baseline parameter and values of systolic arterial pressure variability, and spontaneous baroreflex sensitivity.

	Untrained			Trained			Training factor		Drug factor		Interaction	
	Vehicle	Pyr-5 mg	Pyr-15 mg	Vehicle	Pyr-5 mg	Pyr-15 mg	F_(d.f.)_	*P*	F_(d.f.)_	*P*	F_(d.f.)_	*P*
**Basal parameters**												
Body weight, g	201 ± 4	195 ± 3	200 ± 3	193 ± 3	201 ± 3	215 ± 2	F_(1,53)_: 1.42	0.239	F_(2,53)_: 3.37	0.043	F_(2,53)_: 3.19	0.050
SBP, mmHg	191 ± 1	178 ± 2	166 ± 4	169 ± 1	158 ± 2	136 ± 2	F_(1,53)_: 72.55	< 0.001	F_(2,53)_: 35.95	< 0.001	F_(2,53)_: 1.40	< 0.001
DBP, mmHg	152 ± 3	140 ± 1	130 ± 4	145 ± 2	130 ± 2	109 ± 3	F_(1,53)_: 22.42	< 0.001	F_(2,53)_: 40.75	< 0.001	F_(2,53)_: 2.44	< 0.001
MBP, mmHg	165 ± 2	152 ± 2	142 ± 3	153 ± 1	139 ± 2	118 ± 2	F_(1,53)_: 34.63	< 0.001	F_(2,53)_: 36.54	< 0.001	F_(2,53)_: 1.83	0.172
HR, bpm	379 ± 5	344 ± 8	326 ± 4	350 ± 7	335 ± 7	314 ± 7	F_(1,53)_: 10.20	0.002	F_(2,53)_: 25.22	< 0.001	F_(2,53)_: 2.2	0.233
**SBP variability**												
Variance, mmHg^2^	19 ± 2	26 ± 1	44 ± 2	27 ± 1	34 ± 1	46 ± 2	F_(1,53)_: 5.63	0.022	F_(2,53)_: 27.1	< 0.001	F_(2,53)_: 0.58	0.561
LF, mmHg^2^	6 ± 0.2	8.5 ± 0.3	9.0 ± 0.3	4.7 ± 0.3	9.8 ± 1	13.1 ± 0.3	F_(1,53)_: 11.5	0.001	F_(2,53)_: 68.9	< 0.001	F_(2,53)_: 16.2	< 0.001
**Spontaneous BRS**												
Gain down, ms/mmHg	1.16 ± 0.09	1.12 ± 0.07	1.63 ± 0.06	1.33 ± 0.10	1.41 ± 0.05	1.73 ± 0.06	F_(1,53)_: 12.9	< 0.001	F_(2,53)_: 35.9	< 0.001	F_(2,53)_: 1.53	0.226
Gain up, ms/mmHg	1.21 ± 0.07	1.30 ± 0.08	1.90 ± 0.07	1.16 ± 0.09	1.18 ± 0.04	1.78 ± 0.07	F_(1,53)_: 0.74	0.395	F_(2,53)_: 74.8	< 0.001	F_(2,53)_: 3.12	0.053

All values are presented as the mean ± SEM.

*Pyr-5 mg* pyridostigmine bromide treatment at a dose of 5 mg/kg/day, *Pyr-15 mg* pyridostigmine bromide treatment at a dose of 15 mg/kg/day, *g* gram, *SBP* systolic blood pressure, *mmHg* millimeters of mercury,*DBP* diastolic blood pressure, *MBP* mean blood pressure, *HR* heart rate, *bpm* beats per minute, *LF* low frequency band, *BRS* baroreflex sensitivity, *gain down* baroreflex sequence with progressive decreases in blood pressure followed by progressive decreases in pulse interval, *gain up* baroreflex sequence with progressive increases in blood pressure followed by progressive increases in pulse interval, *F* factor, *df* degrees of freedom.

### Double pharmacological blockade with methylatropine and propranolol-sympathovagal balance

**Figure 2:**
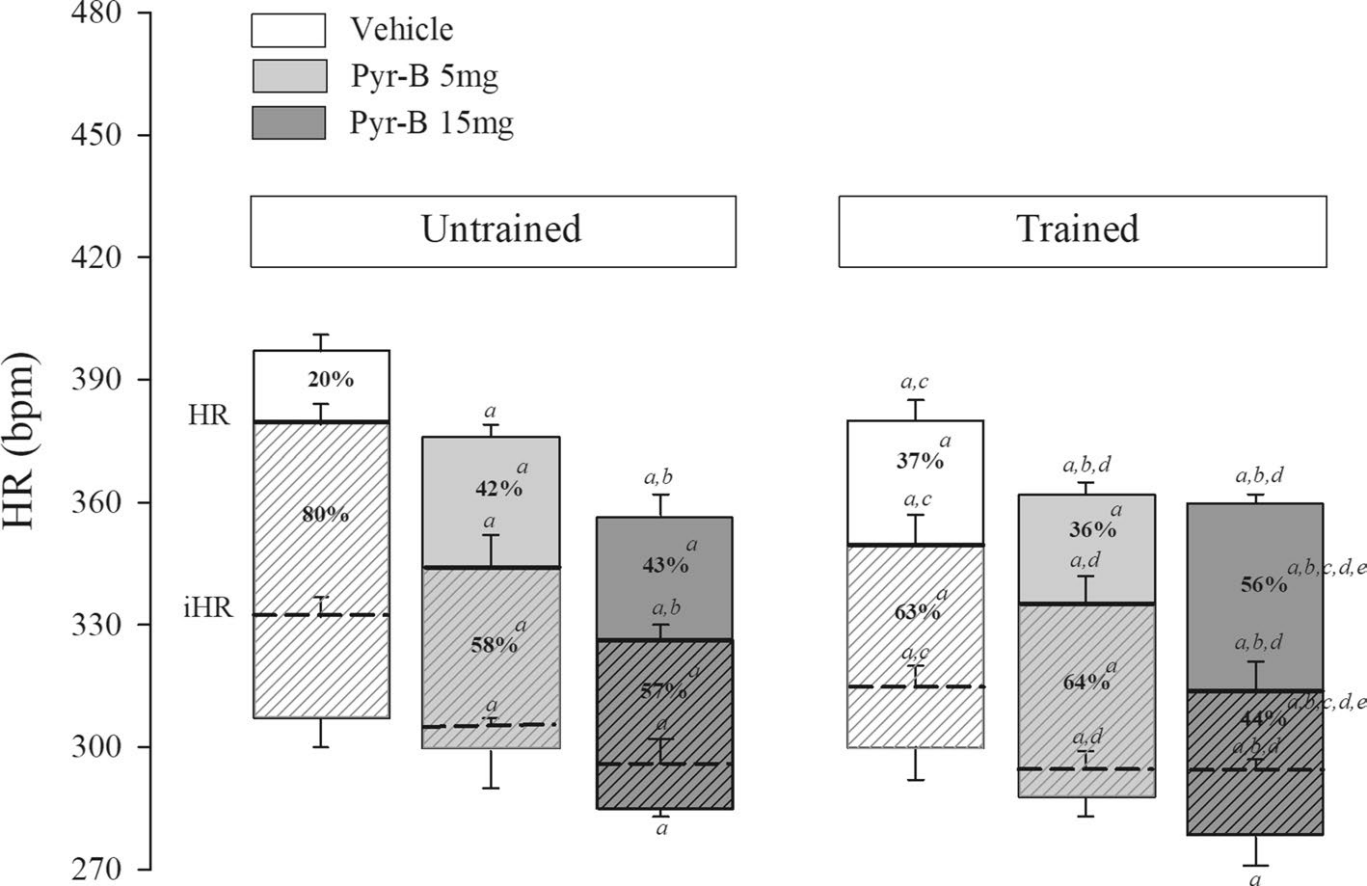
Evaluation of cardiac autonomic control through double pharmacological blockade with methylatropine and propranolol. The bars show the percentage fluctuation of heart rate (HR) and intrinsic heart rate (IHR) after the administration of methylatropine (solid box) and propranolol (cross-hatched box) in vehicle and pyridostigmine bromide (Pyr-B) treatment, both trained and untrained. ^a^Versus untrained SHR vehicle; ^b^versus untrained SHR Pyr-B 5 mg; ^c^versus untrained SHR Pyr-B 15 mg; ^d^versus trained SHR vehicle; ^e^versus trained SHR Pyr-B 5 mg. The figure was drawn in SigmaPlot 11.0 software (Systat Software Inc., San Jose, CA, USA; https://systatsoftware.com).

Figure 2 shows the cardiac autonomic tonic balance seen in the percentage values for each group. Aerobic physical training and treatment with pyridostigmine bromide increased HR response after methylatropine administration and reduced HR response after propranolol administration. This association enhanced the response.

Aerobic physical training and treatment with pyridostigmine bromide reduced the predominance of sympathetic autonomic components in determining baseline HR. However, only the association between physical training and treatment with pyridostigmine bromide (15 mg/kg/day) promoted an inversion in cardiac autonomic tonic balance, which was characterized by a vagal predominance in determining baseline HR. Supplementary Table S1 shows HR values after administration of methylatropine and propranolol as well as the pacemaker IHR values after the administration of both drugs.

### Heart rate variability (HRV)

**Figure 3:**
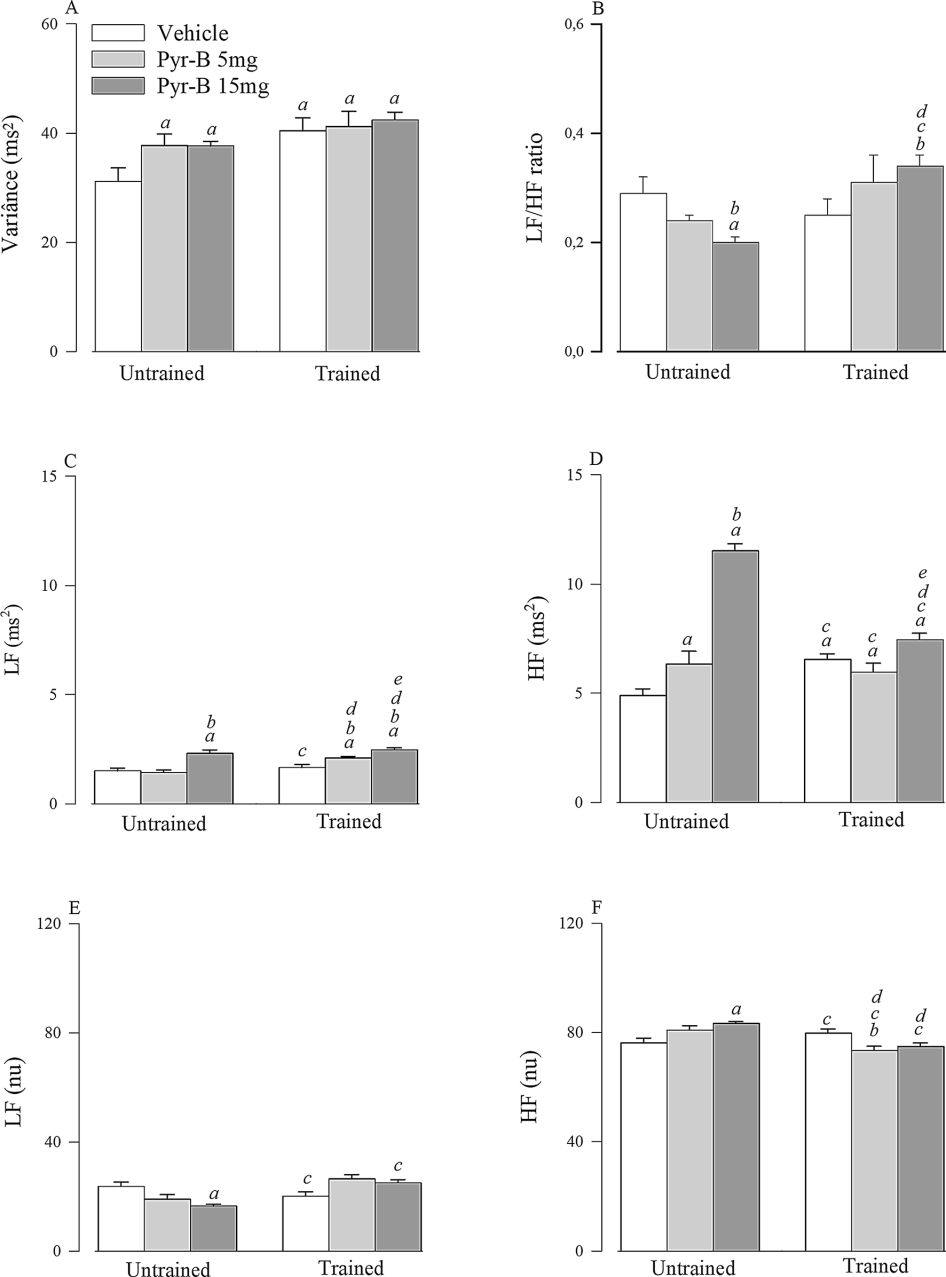
Heart rate variability analysis of all groups before and after the aerobic physical training. All values are presented as mean ± SEM. *Pyr-5 mg* pyridostigmine bromide treatment at a dose of 5 mg/kg/day, *Pyr-15 mg* pyridostigmine bromide treatment at a dose of 15 mg/kg/day, *ms* milliseconds, *LF* low frequency band, *HF* high frequency band, *nu* normalized units. p < 0.05 ^a^versus untrained SHR vehicle; ^b^versus untrained SHR Pyr-B 5 mg; ^c^versus untrained SHR Pyr-B 15 mg; ^d^versus trained SHR vehicle; ^e^versus trained SHR Pyr-B 5 mg. The figure was drawn in SigmaPlot 11.0 software (Systat Software Inc., San Jose, CA, USA; https://systatsoftware.com).

Figure 3 shows intragroup comparison of HRV results. Vehicle group had the lowest values of total variance. The treatment with pyridostigmine bromide with 15 mg/kg/day enhanced oscillation of LF in absolute units and HF in absolute and normalized units, and reduced LF in normalized units and the LF/HF ratio. While the treatment with pyridostigmine bromide with 5 mg/kg/day increased only the oscillations of HF in absolute units. The aerobic physical training increased the total variance of all groups and increased oscillations of HF in absolute units in vehicle group. The association of aerobic physical training with pyridostigmine bromide treatment (5 mg/kg/day) increased oscillations of LF in absolute units and reduced HF in absolute units compared to the pharmacological treatment alone. In turn, the association of aerobic physical training with the higher dosage of pyridostigmine bromide (15 mg/kg/day) reduced the oscillations of HF in absolute and normalized units and LF in normalized units when compared to pyridostigmine bromide treatment alone (15 mg/kg/day). The HRV values are also presented in the Supplementary Table S1.

### Systolic blood pressure variability (BPV) and baroreflex sensitivity (BRS)

Table 1 also shows the BPV and BRS results. While aerobic physical training decreased LF oscillation of BPV, pyridostigmine bromide treatment enhanced it. However, the combination increased the values of LF oscillation even more than the isolated treatment with pyridostigmine bromide. Both aerobic physical training and treatment with pyridostigmine bromide increased BRS to tachycardic responses induced by a reduction in BP (gain down, ms/mmHg). On the other hand, only pyridostigmine bromide treatment enhanced BRS for bradycardic responses induced by an increase in BP (gain up, ms/mmHg).

### Cardiac morphological and functional parameters

The cardiac morphological and functional results obtained using two-dimensional echocardiography are shown in Table 2.

**Table 2:** Values for cardiac function and morphology observed in the untrained and trained groups.

	Untrained			Trained			Training factor		Drug factor		Interaction	
	Vehicle	Pyr-5 mg	Pyr-15 mg	Vehicle	Pyr-5 mg	Pyr-15 mg	F_(d.f.)_	*P*	F_(d.f.)_	*P*	F_(d.f.)_	*P*
**Cardiac morphology**												
RWT, mm/kg	0.5 ± 0.01	0.49 ± 0.02	0.43 ± 0.01	0.5 ± 0.03	0.44 ± 0.01	0.43 ± 0.01	F_(1,53)_: 1.98	0.165	F_(2,53)_: 9.87	< 0.001	F_(2,53)_: 2.6	0.084
IVST, mm/kg	2.16 ± 0.08	1.95 ± 0.1	1.86 ± 0.08	1.93 ± 0.06	1.74 ± 0.08	2.13 ± 0.04	F_(1,53)_: 1.17	0.285	F_(2,53)_: 5.53	0.007	F_(2,53)_: 10.2	< 0.001
LVEDD, mm/kg	32 ± 0.8	31 ± 0.9	32 ± 1.2	31 ± 0.8	34 ± 0.9	34 ± 0.5	F_(1,53)_: 6.4	0.014	F_(2,53)_: 1.28	0.288	F_(2,53)_: 3.6	0.036
LVESD, mm/kg	21 ± 0.7	20 ± 0.1	23 ± 1.2	23 ± 0.9	24 ± 0.8	24 ± 0.8	F_(1,53)_: 22.6	< 0.001	F_(2,53)_: 3.1	0.055	F_(2,53)_: 2.2	0.126
LV mass, mg/g	3.22 ± 0.1	2.91 ± 0.1	3.35 ± 0.3	3.0 ± 0.05	3.11 ± 0.1	3.76 ± 0.1	F_(1,53)_: 2.71	0.106	F_(2,53)_: 16.9	< 0.001	F_(2,53)_: 5.2	0.009
**Cardiac function**												
LVEDV, μL	272 ± 16	264 ± 14	280 ± 17	317 ± 11	312 ± 12	350 ± 22	F_(1,53)_: 54.1	< 0.001	F_(2,53)_: 5.01	0.011	F_(2,53)_: 1.23	0.301
LVESV, μL	65 ± 4	71 ± 5	73 ± 4	85 ± 6	86 ± 4	76 ± 8	F_(1,53)_: 10.6	0.002	F_(2,53)_: 0.43	0.656	F_(2,53)_: 1.79	0.179
Stroke volume, μL	205 ± 19	193 ± 19	208 ± 14	231 ± 22	226 ± 19	275 ± 14	F_(1,53)_: 12.7	< 0.001	F_(2,53)_: 2.75	0.074	F_(2,53)_: 1.21	0.307
Cardiac output, mL/min	63 ± 3	67 ± 5	75 ± 4	72 ± 8	71 ± 5	90 ± 5	F_(1,53)_: 3.5	0.068	F_(2,53)_: 3.73	0.031	F_(2,53)_: 0.41	0.664
Ejection fraction, %	76 ± 1	73 ± 2	74 ± 1	73 ± 1	72 ± 2	79 ± 2	F_(1,53)_: 0.04	0.852	F_(2,53)_: 0.8	0.455	F_(2,53)_: 1.58	0.216
Cardiac index, mL/g	0.31 ± 0.02	0.34 ± 0.02	0.37 ± 0.02	0.36 ± 0.03	0.37 ± 0.02	0.44 ± 0.02	F_(1,53)_: 3.43	0.070	F_(2,53)_: 2.80	0.071	F_(2,53)_: 0.24	0.788

All values are presented as the mean ± SEM.

*Pyr-5 mg* pyridostigmine bromide treatment at a dose of 5 mg/kg/day, *Pyr-15 mg* pyridostigmine bromide treatment at a dose of 15 mg/kg/day, *RWT* relative wall thickness, *mm* millimeter, *kg* kilogram, *IVST* interventricular septum thickness; *LVEDD* left ventricular end-diastolic diameter; *LVESD* left ventricular end-systolic diameter; *LV* left ventricular, *mg* milligram, *g* gram, *LVEDV* left ventricular end-diastolic volume, *μL* microliter, *LVESV* left ventricular end-systolic volume, *mL* milliliter, *min* minute, *%* percentage, *F* factor, *df* degrees of freedom.

While aerobic physical training increased LVEDD and LVESD, pyridostigmine bromide treatment reduced RWT and IVST and enhanced LV mass. The association between aerobic physical training and pyridostigmine bromide treatment, mainly at a dose of 15 mg/kg/day, increased IVST, LVEDD, and LV mass.

Aerobic physical training increased LVEDV, LVESV, and EF while pyridostigmine bromide treatment increased LVEDV and cardiac output. The association between the two treatments did not result in any additional effects. Pyridostigmine bromide treatment at a dose of 15 mg/kg/day promoted an increase in cardiac output and cardiac index compared to the untrained vehicle group. On the other hand, the aerobic physical training groups had higher values of LVEDV and LVESV than the untrained group. The association of aerobic physical training and pyridostigmine bromide, specifically at a dose of 15 mg/kg/day, increased the ejection volume, EF, cardiac output, and cardiac index compared to the trained vehicle group and its respective untrained group.

## Discussion

Aerobic physical training and pyridostigmine bromide treatment had similar effects on cardiac autonomic tonic balance, which were characterized by an increase in vagal influence and/or a reduction in sympathetic influence as well as a reduction in hemodynamic parameters, including BP, baseline HR, and IHR. On the other hand, the association between the two did not enhance these effects.

In SHRs, the accentuated predominance of the sympathetic autonomic drive over the vagal drive is often observed, which contributes to a high baseline HR accompanied by adverse cardiac morphological and functional adaptations^16,28,29^. The reduction in baseline HR, induced only by pyridostigmine bromide treatment, resulted from a greater acetylcholine level. In this case, there is a tendency to reduce the baseline HR-dependent dose; that is, a dose of 15 mg/kg/day promotes greater effects, including in trained animals. Meanwhile, the baseline HR reduction induced by aerobic physical training seems to involve a more complex mechanism, which is characterized by adaptations in the central sites of cardiovascular control^30–32^, downregulation of β-adrenergic receptors^33,34^, and intrinsic cardiac adaptations. According to literature, autonomic adaptations in the central sites of cardiovascular control involve hypothalamic nuclei, which include the paraventricular and supra-optic nuclei and nuclei located in the brainstem such as the nucleus of the solitary tract (NTS) and rostral-ventrolateral medulla (RVLM)^35^. These adaptations seem to involve a series of factors, such as neural remodeling, and the influence of endogenous factors, which result in a decrease in sympathetic autonomic drive and/or an increase in the participation of the vagal autonomic drive in cardiac tonic control^20,36–42^. On the other hand, intrinsic cardiac adaptations also seem to contribute to both baseline HR reduction and IHR reduction, which may be due to cardiac morphological and functional changes.

In our study, we observed that trained SHRs showed an increase in LVEDV and LVESF, resulting in increases in LVEDV and LVESV, as well as an expressive increase in ejection volume. These adaptations probably arise from the greater venous return induced by aerobic physical training. However, we did not observe an increase in cardiac output and cardiac index at rest since the baseline HR reduction offset the greater diastolic filling. Moreover, the echocardiography analysis of the animals treated with pyridostigmine bromide did not show changes in LV diameter but showed modifications in IVST and RWT associated with an increase in LV mass, mainly in animals previously submitted to aerobic physical training. The causes of these morphological adaptations are uncertain, but they suggest that changes in autonomic dynamics induced by aerobic physical training associated with pyridostigmine bromide treatment result in morphological adaptations that favor better cardiac performance. In addition, animals treated with pyridostigmine bromide also showed an increase in cardiac output. This adaptation is also associated with the increase in LVEDV found in these animals, resulting in a tendency to increase the ejection volume (P < 0.074). In addition to the increased cardiac output, the cardiac index (mL/g) remained unchanged, mainly in the 15 mg/kg/day treatment group.

Some studies have related BP reduction to the effects of chronic acetylcholinesterase block on endothelial function and a reduction in oxidative stress, which is also associated with aerobic physical training^43^. However, the mechanisms responsible for BP reduction still need to be further discussed. Besides the aforementioned, other physical training effects can be noted and considered in future studies, including vascular and cardiac sympathetic activity, decreased serum levels of vasoconstrictor factors, and increased endothelial vasodilator factor levels as a result of the enhanced shear stress induced by aerobic physical training, resulting in reduced peripheral vascular resistance^36,44,45^. The causes of the reduction observed after pyridostigmine bromide treatment were potentially more complex. One hypothesis is that pyridostigmine bromide might also block non-neural acetylcholinesterase action produced in other locations such as the endothelium and/or lymphocytes, promoting the hemodynamic changes discussed earlier^9^.

The results of HRV, BPV, and BRS were interesting. Both aerobic physical training and pyridostigmine bromide treatment increased HRV LF oscillations in absolute units. However, only treatment with pyridostigmine bromide, more specifically at a dose of 15 mg/kg/day, increased HRV HF oscillation in absolute units. Until then, we observed that the effects of pyridostigmine bromide treatment on baseline HR, IHR, and cardiac autonomic tonic balance were somewhat similar and that the association with physical training potentiated some responses such as increased vagal tone and reduced baseline HR, BP, and IHR. In contrast, the HRV results showed that the combination of treatments did not potentiate the results obtained with the isolated treatments but attenuated or even reversed the beneficial effects of aerobic physical training and pyridostigmine bromide treatment when applied alone. Regarding HRV, there was a reduction in HF oscillation, which seems to indicate saturation of vagal stimulation from the combination of both treatments^46,47^. Nevertheless, the obtained values were still greater than those obtained for the untrained vehicle group. However, when the autonomic modulation balance represented by the LF/HF ratio was observed, the combination treatment effects were even more evident. This occurrence can be explained by the pyridostigmine bromide effect, which increases both oscillation bands, LF and HF, mainly at a dose of 15 mg/kg/day. The causes of this increase are still uncertain, but they might be associated with an increase in sympathetic modulation oscillation and vagal oscillations since the LF band seems to be mediated by both autonomic components^25^.

As for BPV, the findings were also surprising. While only the trained animals showed a reduction in LF oscillation modulation, the animals treated with pyridostigmine bromide, trained or not, showed a significant enhancement of the LF band. In fact, SHRs are known to have high vascular sympathetic influence, resulting in increased LF oscillations of BPV^48–52^. The cause of this increase remains unknown and cannot be attributed to baroreflex malfunction even in animals treated only with pyridostigmine bromide since the BRS was increased. This statement is based on the concept that baroreflex is one of the main mechanisms involved in LF oscillations of BPV alongside vascular sympathetic drive^53^ and relaxing factors derived from the vascular endothelium such as nitric oxide (NO). The baroreflex and NO would act as a buffer system for BP fluctuations induced by sympathetic activation^54^. Therefore, some authors suggest that the increase in LF oscillation of BP is related to a decrease in BRS and a decrease in both NO production and release. However, a study has demonstrated that long-term administration of acetylcholinesterase inhibitors, pyridostigmine or donepezil, attenuates vascular reactivity dysfunction in SHRs by decreasing reactive oxygen species generation and increasing NO bioavailability, possibly via increased endothelial NO synthase activity and inhibition of nicotinamide adenine dinucleotide phosphate (NADPH) oxidase activity^43^. As noted, the explanation of the effects of pyridostigmine bromide on vascular modulation is even more complex than that of the effects on cardiac control. However, both require further investigation.

Treatment with pyridostigmine bromide, mainly at a dose of 15 mg/kg/day, appears to be beneficial to cardiac autonomic regulation and function parameters; it increased vagal autonomic tonic influence in determining baseline HR and increased HRV and BRS. In turn, the enhancement of the BPV is worrying. The association with aerobic physical training potentiates the reduction in hemodynamic parameters and results in a greater vagal autonomic tonic influence on the heart. However, this combination reduces vagal modulation of the heart. The mechanisms involved in these findings remain uncertain and require further investigation.

## Supplementary Information

Supplementary Information.

## Data Availability

All data used during the current study are included in this published article or are available from the corresponding author upon reasonable request.
