# Supplementary material for: Effects of chronic cholinergic stimulation associated with aerobic physical training on cardiac morphofunctional and autonomic parameters in spontaneously hypertensive rats
Source: Sci Rep. 2021 Aug 25;11:17141. doi: 10.1038/s41598-021-96505-2 (PMC8387354; doi:10.1038/s41598-021-96505-2)
Supplement: Supplementary file 1 — Supplementary Information. [file 41598_2021_96505_MOESM1_ESM.pdf]

Effects of chronic cholinergic stimulation associated with aerobic physical training on cardiac morphofunctional and autonomic parameters in spontaneously hypertensive rats

Camila B. Gardim[1]; Ana Catarine V. Oliveira[1]; Bruno A. Aguilar[1]; Stella V. Philbois[1], Hugo C. D. Souza[1*]

[1] Ribeirão Preto Medical School, University of São Paulo, Ribeirão Preto, SP, Brazil

*Corresponding author: hugocds@fmrp.usp.br

Results

Aerobic physical training increased the total variance, oscillation of LF in absolute units, and LF/HF ratio. In turn, pyridostigmine bromide treatment increased the oscillation of both LF and HF in absolute units. On the other hand, the association of both to aerobic physical training attenuated the oscillation of HF in absolute units and increased the LF/HF ratio.

Table S1. Values of cardiac autonomic tonic balance after the pharmacological blockade with methylatropine and propranolol and heart rate variability.

	UNTRAINED			TRAINED			Training Factor		Drug Factor		Interaction	
	**Vehicle**	**Pyr-5mg**	**Pyr-15mg**	**Vehicle**	**Pyr-5mg**	**Pyr-15mg**	$F_{(d.f.)}$	***P***	$F_{(d.f.)}$	***P***	$F_{(d.f.)}$	***P***
**Autonomic tonic control**												
HR / methylatropine, bpm	397 ± 4	376 ± 3	357 ± 5	380 ± 5	362 ± 3	360 ± 2	$F_{(1,53)}$: 7.75	0.008	$F_{(2, 53)}$: 26.03	<0.001	$F_{(2,53)}$: 3.15	0.052
Δ HR / methylatropine, bpm	18 ± 2	32 ± 1	31 ± 3	30 ± 1	27 ± 1	46 ± 4	$F_{(1,53)}$: 18.21	<0.001	$F_{(2, 53)}$: 23.39	<0.001	$F_{(2,53)}$: 13.99	<0.001
HR / propranolol, bpm	307 ± 7	299 ± 9	285 ± 2	300 ± 8	288 ± 5	278 ± 7	$F_{(1,53)}$: 4.23	0.045	$F_{(2, 53)}$: 8.97	<0.001	$F_{(2, 53)}$: 0.08	0.925
Δ HR / propranolol, bpm	72 ± 5	45 ± 7	41 ± 5	50 ± 2	47 ± 8	36 ± 5	$F_{(1,53)}$: 7.89	0.007	$F_{(2, 53)}$: 21.17	<0.001	$F_{(2, 53)}$: 5.61	0.006
IHR, bpm	331 ± 5	305 ± 2	296 ± 6	315 ± 5	295 ± 4	295 ± 2	$F_{(1,53)}$: 5.75	0.002	$F_{(2, 53)}$: 17.49	<0.001	$F_{(2, 53)}$: 1.29	0.283
**Heart rate variability**												
PI, ms	158 ± 2	174 ± 5	184 ± 2	171 ± 3	179 ± 3	191 ± 5	$F_{(1,53)}$: 4.79	0.033	$F_{(2,53)}$: 12.06	<0.001	$F_{(2,53)}$: 0.36	0.700
Variance, ms^2	30 ± 2	37 ± 2	37 ± 1	40 ± 3	42 ± 3	42 ± 2	$F_{(1,53)}$: 6.44	0.014	$F_{(2,53)}$: 1.12	0.334	$F_{(2,53)}$: 0.38	0.687
LF, ms^2	1.5 ± 0.1	1.4 ± 0.1	2.3 ± 0.2	1.7 ± 0.1	2.1 ± 0.06	2.5 ± 0.1	$F_{(1,53)}$: 8.76	0.005	$F_{(2,53)}$: 15.8	<0.001	$F_{(2,53)}$: 1.82	0.173
LF, nu	21 ± 2	21 ± 3	17 ± 0.6	22 ± 0.7	20 ± 3	25 ± 2	$F_{(1,53)}$: 2.36	0.131	$F_{(2,53)}$: 0.14	0.874	$F_{(2,53)}$: 3.10	0.054
HF, ms^2	4.5 ± 0.3	5.6 ± 0.4	11 ± 0.3	6.6 ± 0.4	5.7 ± 0.5	7.0 ± 0.4	$F_{(1,53)}$: 3.74	0.059	$F_{(2,53)}$: 52.3	<0.001	$F_{(2,53)}$: 32.2	<0.001
HF, nu	79 ± 2	79 ± 3	83 ± 0.6	78 ± 1	80 ± 3	74 ± 2	$F_{(1,53)}$: 1.66	0.204	$F_{(2,53)}$: 0.1	0.906	$F_{(2,53)}$: 1.22	0.304
LF/HF ratio	0.29 ± 0.03	0.24 ± 0.01	0.20 ± 0.01	0.25 ± 0.03	0.31 ± 0.05	0.34 ± 0.02	$F_{(1,53)}$: 5.86	0.019	$F_{(2,53)}$: 0.06	0.945	$F_{(2,53)}$: 5.72	0.006

All values are presented as the mean ± SEM. Pyr-5 mg, pyridostigmine bromide treatment at a dose of 5 mg/kg/day; Pyr-15 mg, pyridostigmine bromide treatment at a dose of 15 mg/kg/day bpm, beats per minute; HR, heart rate; Δ HR, heart rate variation delta; IHR, intrinsic heart rate; PI, pulse interval; ms, milliseconds; LF, low frequency band; nu, normalized units; HF, high frequency band; F, factor; df, degrees of freedom.
